# ACTL6A expression promotes invasion, metastasis and epithelial mesenchymal transition of colon cancer

**DOI:** 10.1186/s12885-018-4931-3

**Published:** 2018-10-22

**Authors:** Zhijun Zeng, Hao Yang, Shuai Xiao

**Affiliations:** 1 Department of Geratic Surgery, Central South University, Xiangya Road 87, Changsha, Hunan 410008 People’s Republic of China; 2 National Clinical Research Center for Geriatric Disorders, Xiangya Hospital, Central South University, Xiangya Road 87, Changsha, Hunan 410008 People’s Republic of China

**Keywords:** ACTL6A, Colon cancer, Invasion, Metastasis, EMT

## Abstract

**Background:**

Metastasis is the main cause of death in patients with advanced stage colon cancer. Epithelial mesenchymal transition (EMT) plays an important role in invasion and metastasis. Actin-like 6A (ACTL6A) is vital for embryogenesis and differentiation and is also critical for metastasis and EMT in hepatocellular carcinoma, as observed in our previous study. In the present study, we further explored the role of ACTL6A in colon cancer metastasis.

**Methods:**

ACTL6A expression levels were analyzed in normal colon, colon adenoma and colon cancer specimens using public databases and tissue samples. ACTL6A expression and its association with clinicopathologic features of colon cancer patients were also analyzed. ACTL6A-overexpression and ACTL6A-knockdown colon cancer cells were used to perform cytological experiments to explore the potential biological function of ACTL6A in metastasis and EMT in colon cancer.

**Results:**

The data from both the Gene Expression Omnibus (GEO) and Oncomine databases showed that ACTL6A expression levels in colon adenoma and cancer were higher than those in normal colon samples. The ACTL6A expression level in fresh colon cancer specimens was also higher than that in the corresponding adjacent normal colon specimens. Patients with high ACTL6A expression directly correlated with advanced pT status, distant metastasis, poor differentiation and microvascular/perineural invasion. ACTL6A overexpression promoted migration and invasion of colon cancer cells, whereas ACTL6A knockdown exhibited the opposite effect in vitro. Moreover, we demonstrated that ACTL6A promoted EMT in colon cancer cells in vitro.

**Conclusions:**

Our findings indicate that ACTL6A exhibits pro-tumor function and acts as an EMT activator in colon cancer. ACTL6A may serve as a potential therapeutic target for colon cancer.

**Electronic supplementary material:**

The online version of this article (10.1186/s12885-018-4931-3) contains supplementary material, which is available to authorized users.

## Background

Colon cancer remains one of the most common and deadly cancers worldwide in the twenty-first century [1]. In the last 2 decades, within imaging techniques and therapeutic strategies advance, the 5-year survival rate for all stages of colon cancer has increased to 66%. However, the survival rate for distant-stage colon cancer is still very poor, the 2-year survival rate is only approximately 20–30% mainly due to metastasis [1, 2]. Thus, a good understanding of the metastasis process and mechanism is very important for colon cancer.

Metastasis is a multistep invasion metastasis cascade, composed of local invasion, intravasation, survival in the circulation, arrest at a distant organ site, extravasation, initial survival in a foreign microenvironment and micrometastasis formation, and finally metastatic colonization. Increasing evidence has suggested that epithelial mesenchymal transition (EMT) plays an important role in this pathological process, especially in the initiation of local invasion [3]. EMT is a complex cell-biological program that endows epithelial cells with mesenchymal characteristics, and it is critical for embryonic morphogenesis and tissue repair. Importantly, EMT enhances cell motility, migration, invasiveness and resistance to apoptosis, which endows cancer cells with invasion and metastasis potential and, finally, promotes cancer progression and metastasis [4]. Accumulating evidence strongly indicates that EMT participates in the metastasis of various epithelial tumors including colon cancer. Thus, thorough comprehension of the EMT regulatory mechanism is crucial in controlling colon cancer metastasis.

EMT is mainly driven by EMT-activating transcription factors (EMT-TFs) [5]. Actin-like 6A (ACTL6A, also known as BAF53A) is a chromatin-remodeling factors, which encodes an SWI/SNF subunit, mainly transcriptionally regulating stem cell and progenitor cell function, as observed in initial studies [6, 7]. Our previous study has found that ACTL6A acted as an important oncogenic driver and novel EMT-TF in hepatocellular carcinoma (HCC), as well as was associated with prognosis of HCC [8]. Besides, studies have found that ACTL6A played an central oncogenic role in progression and metastasis in cancers such as squamous cell carcinoma (SCC), rhabdomyosarcoma and glioma [9–11]. However, the role of ACTL6A in colon cancer is still unknown.

In this study, we intend to explore the functional role of ACTL6A in colon cancer, elucidate its association with EMT, and finally exploit its potential clinical value.

## Methods

### Public databases

The public database Gene Expression Omnibus (GEO) (https://www.ncbi.nlm.nih.gov/geoprofiles/) and Oncomine (https://www.oncomine.org) were used respectively for the analysis of ACTL6A expression in normal colon, colon adenoma and cancer. The search terms were “ACTL6A and colon cancer” in GEO database. The “Gene: ACTL6A”, “Analysis Type: Cancer vs. Normal Analysis”, “Cancer Type: Colon Adenocarcinoma”, “Cancer Type: Colon Adenoma” and “Data Type: mRNA” were the search terms in Oncomine database.

### Patients and samples

20 paired of randomly selected fresh colon cancer and adjacent normal tissues were collected after radical resection in the department of geratic surgery of Xiangya Hospital, and then stored in Ultra-low temperature refrigerator at − 80 °C. 92 cases of randomly selected paraffin-embedded colon cancer tissue were obtained and sliced with 4 μm section. All the samples came from the geratic surgery of Xiangya Hospital from Jan 2016 to Dec 2017, and were pathology confirmed by 2 independent pathologists. All patients have signed written informed consent, and the clinicopathological data were collected by a specially-assigned member. The study was approved by the ethics committee of Xiangya Hospital of Central South University (CSU) which was accordance with the Declaration of Helsinki.

### Real-time PCR

TRIzol® Reagent (Life Technologies, Carlsbad, CA) was used to isolate total ribonucleic acid (RNA) using the universal complementary deoxyribonucleic acid (cDNA) synthesis kit (Toyobo, Tokyo, JP). The RNA was then reverse-transcribed to obtain cDNA by the universal cDNA synthesis kit (Toyobo, Tokyo, JP) at 37 °C for 50 min. Real-time PCR was performed using the SYBR Green Realtime PCR Master Mix (Toyobo, Tokyo, JP) as previous described [8]. All quantifications were normalized to the level of endogenous GAPDH as a control. The ACTL6A primers were as follows: forward, 5′ - CCAGGTCTCTATGGCAGTGTAA -3′ and reverse, 5′ - CGTAAGGTGACAAAAGGAAGGTA -3′; GAPDH primers were: forward, 5′ - GTCTCCTCTGACTTCAACAGCG -3′ and reverse, 5′ - ACCACCCTGTTGCTGTAGCCAA -3′.

### Western-blot

Total proteins of cells were extracted with RIPA lysis buffer (Thermo Scientific, Waltham, MA).) and separated by SDS-PAGE and then transferred to the polyvinylidene fluoride (PVDF) membrane (Millipore, Bedford, MA). The membrane were blocked with 5% skimmed milk and incubated with the appropriate antibody. The antigen-antibody complex on the membrane was detected with enhanced chemiluminescence regents (Thermo Scientific, Waltham, MA). The antibodies and dilution were as follows: ACTL6A (sc-137,062, dilution 1:1000, Santa Cruz Biotechnology, Santa Cruz,CA), β-actin (A5316, dilution 1:3000, Sigma Aldrich, St. Louis, MO), E-cadherin (sc-7870, dilution 1:1500, Santa Cruz Biotechnology, Santa Cruz,CA), vimentin(sc-7557, dilution 1:1000, Santa Cruz Biotechnology, Santa Cruz,CA).

### Immunohistochemistry

Immunohistochemical staining for tissue was performed using the polymer HRP detection system (Zhongshan Goldenbridge Biotechnology) on formalin-fixed, paraffin-embedded, 4 μm thickness tissue sections. The paraffin sections were dewaxed, antigen retrieval, block endogenous peroxidase, donkey serum, primary antibodies incubation, HRP conjugated secondary antibody incubation, DAB staining, counterstained by hematoxylin, dehydrated in sequence as previous described [8]. The dilution of primary antibody of ACTL6A (sc-137,062) was 1:300. The immunohistochemical staining scores were also observed and recorded as previous described [8].

### Immunofluorescence staining

The cell immunofluorescence was according to the protocol of Abcam. Cells growth at glass coverslips, and then fixed with 4% paraformaldehyde in phosphate-buffered saline (PBS) with 0.2% Triton. Next, cells were blocked for an hour with 1% bovine serum albumin followed by incubation with primary antibody (E-cadherin, dilution 1:600) overnight at 4 °C, then washed and incubated with appropriate secondary antibody (Donkey anti-Mouse IgG (H + L) cross adsorbed secondary antibody, DyLight 594 conjugate, SA5–10168, dilution 1:1000, Thermo Scientific, Waltham, MA) and DAPI as previous described [8].

### Cell lines and lentiviral transduction

The human colon cancer cell lines SW480 and SW620 were kindly provided by Stem Cell Bank, Chinese Academy of Sciences (Shanghai, China) (Additional file 1). Cell culture was according to the manufacturer’s protocol and growed at 37 °C with 5% CO2. The ACTL6A ectopic expression and knockdown lentivirus and their negative control (NC) lentivirus were purchased from GeneChem (Shanghai, China). Full-length human ACTL6A ectopic expression lentivirus were transfected into SW480 cells, and short hairpin RNAs (shRNA) lentivirus targeting ACTL6A were transfected into SW620 cells according to the manufacturer’s instructions. Cells transfected with empty vector were used as controls. Enhanced Infection Solution (ENi.S., GeneChem, Shanghai, China) and 5 μg/mL Polybrene (GeneChem, Shanghai, China) were used to enhance the transfection efficiency to obtain stably transfected cells as described [12]. The ACTL6A RNAi sequences were as follow: RNAi-1: 5’-TCCAAGTATGCGGTTGAAA-3′; RNAi-2: 5’-GTACTTCAAGTGTCAGATT-3′; RNAi-3: 5’-GGGATAGTTTCCAAGCTAT-3′. The interfered efficiency was detected by real-time PCR (Additional file 2: Figure S1A), and RNAi-1 was chose for further assays.

### Wound-healing assay

Cells were cultured in six-well plates containing DMEM with 10% FBS. When cells grew to 90% confluence, they were pre-incubated with Mitomycin-C (10 μg/ml) for 1 h at 37 °C to suppress cell proliferation, next rinsed with PBS, and then starved for 24 h in serum-free medium. A sterile 10 μL pipette tip was used to create wounds, and areas of wound lines were observed and assessed by inverted microscope after 24 h. These experiments were performed in triplicate.

### Transwell migration and invasion assays

The transwell migration assay was conducted as following: the cultured cells were pre-incubated with Mitomycin-C (10 μg/ml) for 1 h at 37 °C to suppress proliferation, then 2 × 10^4^ cells in serum-free medium containing 0.1% bovine serum albumin were placed into the upper chamber of the insert and 500 μl complete medium were added into the bottom chamber. After 12 h of incubation cells were washed by PBS, the cells adhering to the lower membrane of the inserts was counted after staining with 0.1% crystal violet. The numbers of cells was counted under an inverted microscope. The transwell invasion assay was conducted as former, but the upper chamber was paved with Matrigel (5 mg/ml, BD Biosciences, MA). These experiments were also performed in triplicate.

### Statistical analysis

All of the statistical analyses were performed by SPSS 17.0 (SPSS Inc., Chicago, IL) software and the statistical graphs were generated by GraphPad Prism 6.0 (GraphPad Software, Inc., La Jolla, CA). The continuous data were presented as mean ± standard deviation. The comparison of quantitative data between two groups was used Student’s *t*-test, the multiple comparisons were used one-way ANOVA test. The comparison of categorical data was analyzed by the Chi-square test or Fisher exact test. A two-tailed *P* value of less than 0.05 was considered statistical significance. More methods details are available in the Additional file 1. 

## Results

### ACTL6A expression was upregulated in colon cancer from public databases

**Fig. 1:**
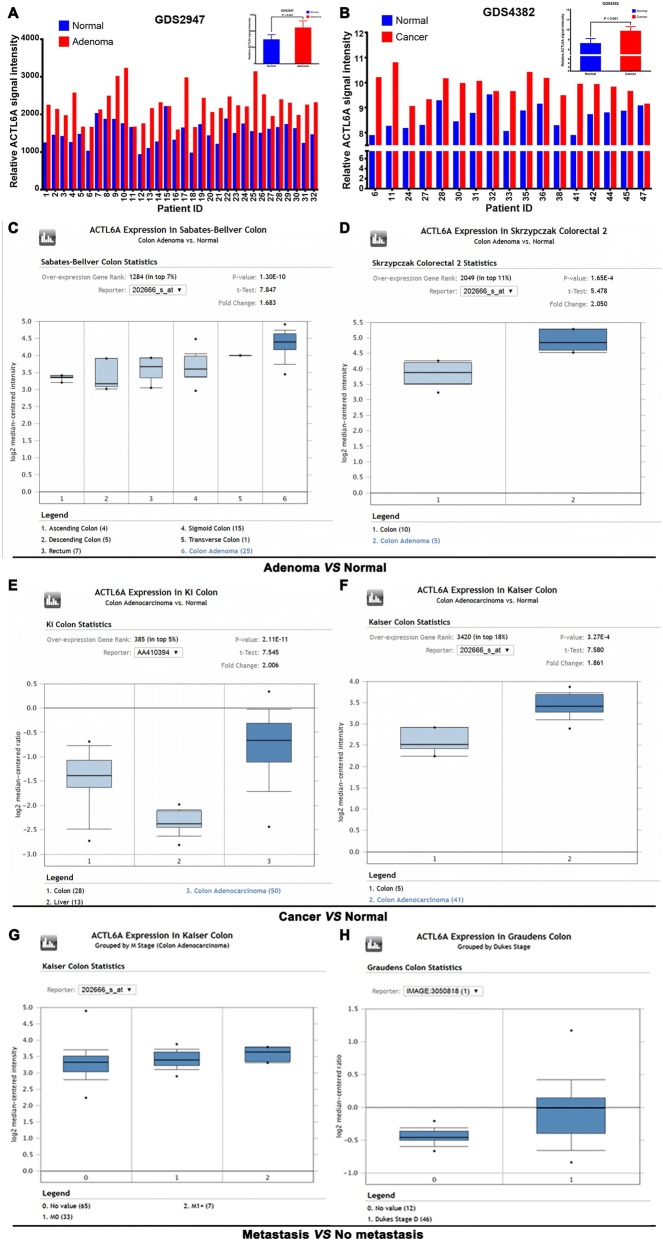
ACTL6A expression was upregulated in colon cancer from public databases. **a** Data of GDS2947 from the GEO database showed that relative ACTL6A expression level in 32 cases of colon adenoma was significantly higher than that in the corresponding normal colon tissue (*P* < 0.001). **b** Data of GDS4382 also showed that the ACTL6A expression level in 17 pairs of colon cancer was significantly higher than that in the corresponding normal colon tissue (*P* < 0.001). **c-d** Data of Sabates-Bellver’s colon (**c**) and Skrzypczak’s colon (**d**) from the Oncomine database showed that the ACTL6A mRNA expression level in colon adenoma was significantly higher than that in normal colon tissue (P < 0.001, respectively). (E-F) Data from Ki’s colon (**e**) and Kaiser’s colon (**f**) showed that ACTL6A expression in colon cancer was obviously higher than that in normal colon tissue (*P* < 0.001, respectively). **g** Data from Kaiser’s colon showed that ACTL6A expression in M1 stage was higher than that in no metastasis group. **h** Data from Graudens’ colon showed that ACTL6A expression in Dukes D stage was higher than that in without distant metastasis group

We first explored ACTL6A expression in colon adenoma and colon cancer using the GEO database by searching “ACTL6A and colon cancer”. In GDS2947 of the GEO database, 32 pairs of human colonic normal mucosa and adenoma biopsy specimen were analyzed using Affymetrix Microarray Suite version 5.0. Results showed that the ACTL6A expression in colon adenoma was significantly higher than that in normal mucosa (Fig. 1a, *p* < 0.001). Seventeen pairs of cancer and noncancerous tissues from surgically resected colon cancer were analyzed in another GEO database GDS 4382; the results showed that the ACTL6A signal intensity in the cancer tissue was significantly higher than that in the paired noncancerous tissue (Fig. 1b, *p* < 0.001). Next, we also detected ACTL6A mRNA expression in the Oncomine database and found that ACTL6A expression in colon adenoma was significantly higher than that in normal colon according to Sabates-Bellver’s data (Fig. 1c) and Skrzypczak’s data (Fig. 1d) (*P* < 0.001, respectively). Next, ACTL6A expression in colon cancer was also detected, which was consistent with the previous results that ACTL6A expression was higher in colon carcinoma than in normal colon tissue in Ki’s data (Fig. 1e) and Kaiser’s data (Fig. 1f) (*P* < 0.001, respectively). We further searched ACTL6A expression with the metastasis of colon cancer in Oncomine database, results showed ACTL6A expression in M1 stage (Fig. 1g) or Dukes D stage (Fig. 1h) group was higher than group without metastasis. These public databases results indicated that ACTL6A expression was upregulated in colon cancer tissue and indicated that ACTL6A might be act as an oncogenic role and associated progression of colon cancer, which was worthy of further study.

### ACTL6A expression was upregulated in colon cancer tissues

**Fig. 2:**
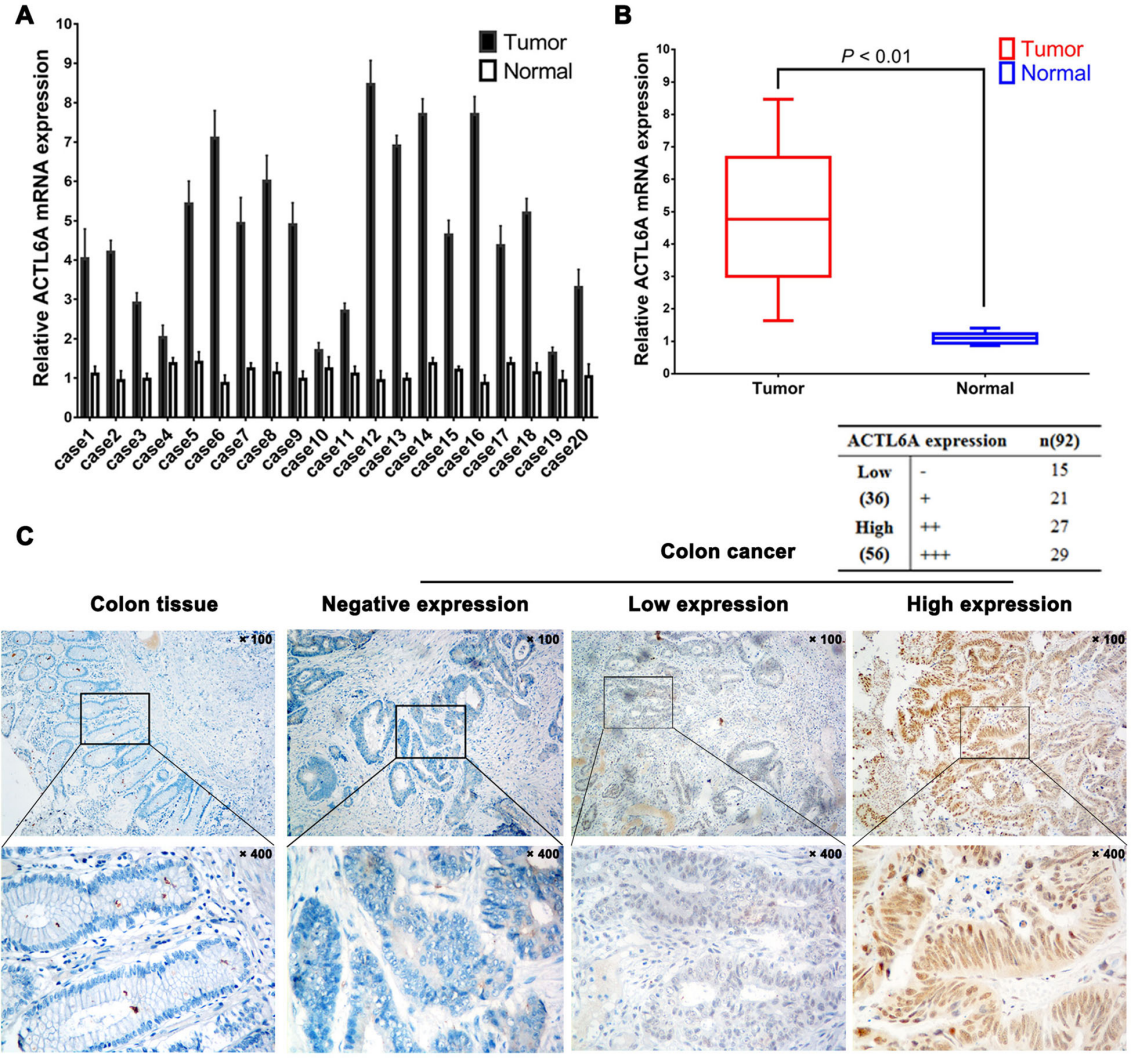
ACTL6A expression was upregulated in colon cancer tissue. **a-b** The results from real-time PCR showed that ACTL6A expression in 20 pairs of colon cancer was higher than that in the corresponding adjacent normal colon tissue (**a**), and statistical analysis also identified that ACTL6A expression in colon cancer group was significant than normal group (**b**) (*P* < 0.01). **c** The representative photographs for immunohistochemical staining of ACTL6A in normal colon tissue (the left panel) and colon cancer (right 3 panels); figures showed ACTL6A was negative expressed in normal colon tissue (the left panel), and ACTL6A positive staining mainly in the nuclei of colon cancer cells, the right 3 panels showed negative, low or high ACTL6A expression in colon cancer

Next, ACTL6A mRNA expression in 20 pairs of fresh colon cancer and noncancerous tissues from our center was detected via real-time PCR. The results showed that the ACTL6A mRNA expression level was higher in cancer tissues than in noncancerous tissues (*P* < 0.01, Fig. 2a, b). Moreover, the ACTL6A protein expression in colon cancer was detected via immunochemistry in 92 cases of paraffin-embedded colon cancer tissues. The data indicated that most of specimens (74 cases, 80.4%) exhibited positive ACTL6A staining, 56 of the cases (60.9%) exhibited relatively high ACTL6A expression (moderate to strong positive staining), and 36 cases exhibited relatively low ACTL6A expression (mild or no positive staining), and ACTL6A expression in normal colon tissue was absent (Fig. 2c). These results indicated that ACTL6A expression was upregulated in colon cancer and might be a biomarker for colon cancer.

### ACTL6A expression was associated with the adverse clinicopathological features of colon cancer

Next, we explored the association of the ACTL6A protein expression level with the clinicopathological features of 92 cases of colon cancer patients. Interestingly, ACTL6A expression was positively correlated with advanced pT status (*P* = 0.007), distant metastasis (*P* = 0.023), poor differentiation (*P* = 0.014) and microvascular/perineural invasion (*P* = 0.002) (Table 1). However, ACTL6A expression exhibited no significant relevance with gender, age, tumor size, tumor location, pN status and mismatch repair (MMR) status (all *P* > 0.05, respectively). These results indicated that high ACTL6A expression was positively correlated with adverse clinicopathological features especially factors related with metastasis, which further suggested that ACTL6A expression was associated with invasion and metastasis of colon cancer.

**Table 1:** The correlation between ACTL6A expression and clinicopathologic features of colon cancer patients

Variables	Total	ACTL6A expression		*P*
	n(92)	Low(36)	High(56)	
Gender				0.524
Female	41	15	26	
Male	51	22	29	
Age (year)				0.641
≤ 60	45	17	28	
>60	47	20	27	
Tumor size(cm)				0.518
≤ 5	51	19	32	
>5	41	18	23	
Tumor location				
Right colon	57	25	32	0.363
Left colon	35	12	23	
pT status				0.007
pT1–2	44	24	20	
pT3–4	48	13	35	
pN status				0.371
pN0	40	14	26	
pN1–2	52	23	29	
Distant metastasis				0.023
Absence	77	35	42	
Presence	15	2	13	
Differentiation				0.014
Well/moderate	53	27	26	
Poor	39	10	29	
Microvascular/perineural invasion iinininvasion				0.002
Absence	60	31	29	
Presence	32	6	26	
MMR status				0.390
Proficient	78	33	45	
Deficiency	14	4	10	

### ACTL6A promoted colon cancer cell migration and invasion in vitro

**Fig. 3:**
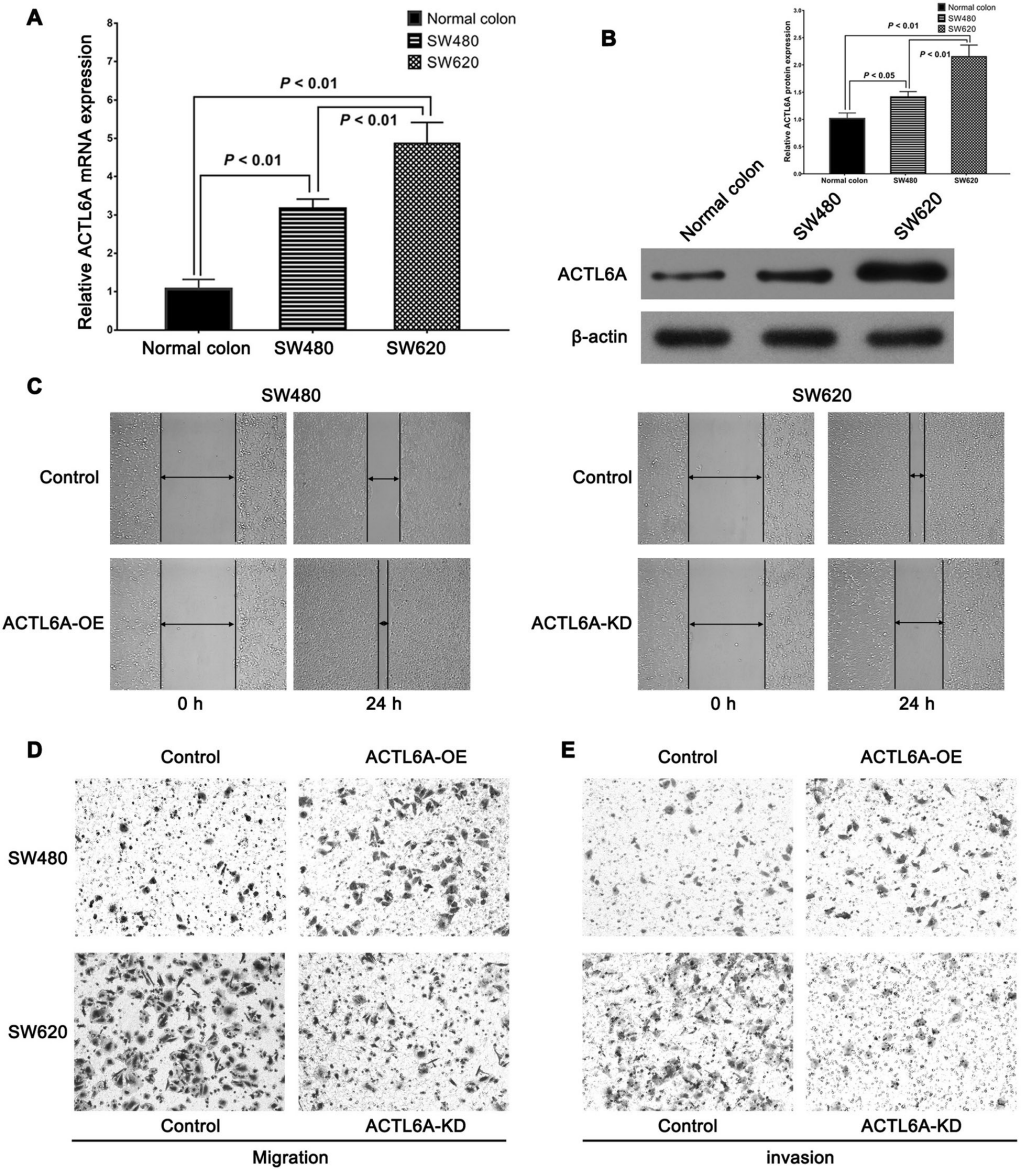
ACTL6A promoted migration and invasion of colon cancer cells. **a-b** ACTL6A expression in colon cancer cell lines showed that the ACTL6A mRNA (**a**) and protein (**b**) expression level in SW620 cells were higher than that in SW480 cells. **c** Wound-healing assay showed that ACTL6A overexpression increased wound healing rate of SW480 cells, while ACTL6A knockdown decreased the wound healing rate of SW620 cells. **d** Migration assay showed that ACTL6A overexpression increased the migration ofSW480 cells, and vice versa, ACTL6A knockdown decreased it. **e** Invasion assay showed that ACTL6A overexpression enhanced the migration of SW480 cells to the Matrigel membrane, but ACTL6A knockdown reduced the effect

We chose SW480 and SW620 cells for further study, because they came from the primary lesion and metastatic lymph node of the same colon cancer patient. Real-time PCR and western-blot results showed that the cell lines exhibited different ACTL6A expression levels (Fig. 3a, b). Next, we constructed ACTL6A knockdown SW620 (SW620^ACTL6A-KD^) and ACTL6A ectopic overexpression SW480 (SW480^ACTL6A-OE^) cell lines to study the functional role of ACTL6A in colon cancer cells in vitro. The results from the wound-healing assay and transwell migration assay showed that the ectopic overexpression of ACTL6A could obviously enhance the wound-healing rate and number of penetrable cells of SW480 cells. Conversely, ACTL6A knockdown in SW620 cells had the significant opposite effect (Fig. 3c-d). These assays indicated that ACTL6A overexpression promoted the migration capacity of colon cancer cells. Accordingly, transwell invasion assays also showed that more SW480 cells traversed the cell-permeable membrane with the ectopic overexpression of ACTL6A, while less SW620 cells traversed the membrane with ACTL6A knockdown (Fig. 3e), which indicated that ACTL6A promoted the invasiveness of colon cancer cells. Combined these results, the study indicated that ACTL6A overexpression promoted the migration and invasion capacity of colon cancer cells, and, thereby, could potentially promote the progression and metastasis of colon cancer.

### ACTL6A induced EMT in colon cancer cells in vitro

**Fig. 4:**
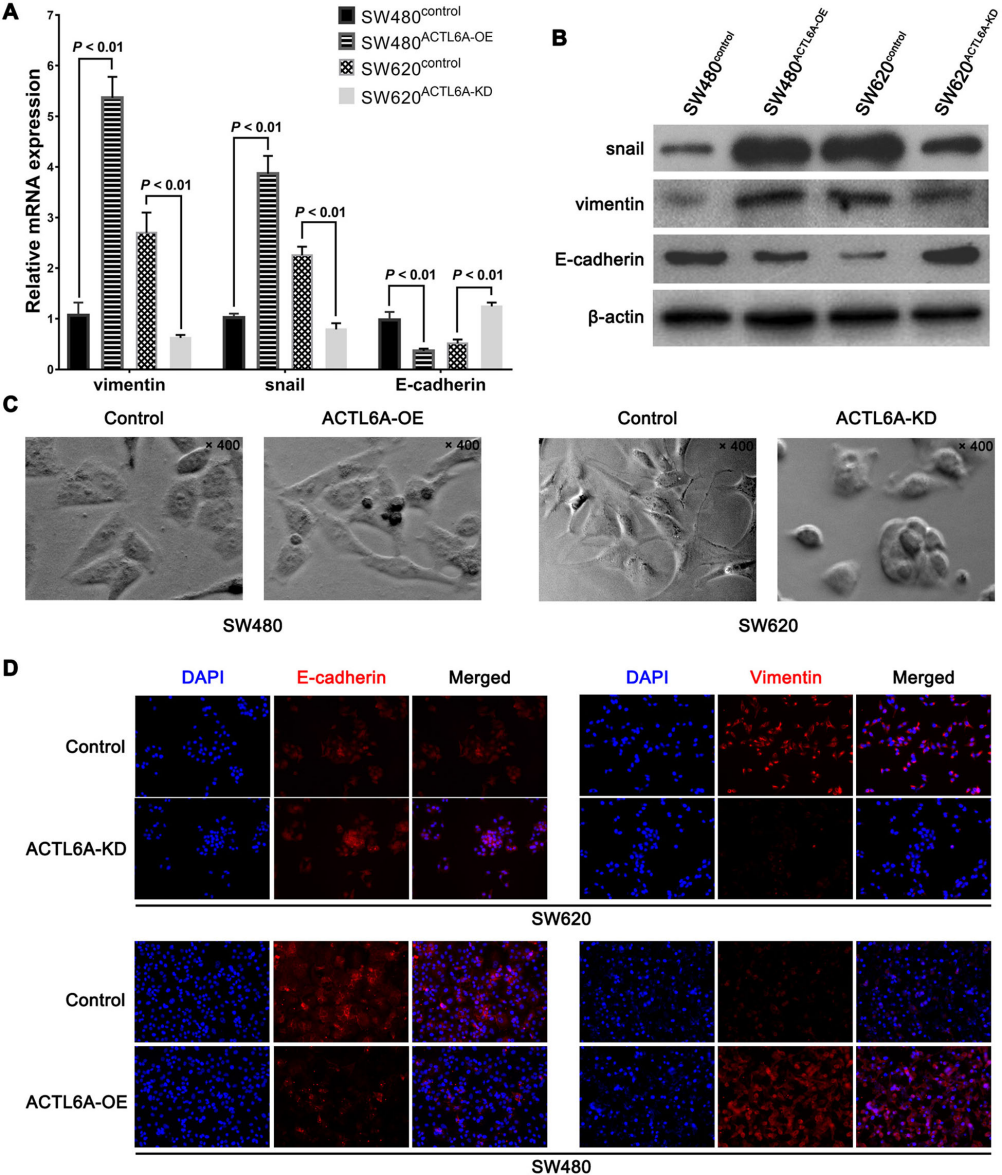
ACTL6A promoted epithelial mesenchymal transition of colon cancer cells. **a-b** Real-time PCR (**a**) and western-blot results (**b**) showed that the expression level of the mesenchymal marker vimentin and snail were upregulated when ACTL6A was overexpressed but was downregulated when ACTL6A was knocked down; and vice versa, epithelial marker E-cadherin was upregulated when ACTL6A was knocked down but was downregulated when ACTL6A was overexpressed. **c** Analysis of the cell morphology showed that SW480 cells were more elongated when ACTL6A was overexpressed, while SW620 cells changed to an oval shape when ACTL6A was knocked down. **d** Cell immunofluorescence assay showed that ACTL6A knockdown induced a stronger E-cadherin and weaker Vimentin expression in SW620 cells, while ACTL6A overexpression in SW480 cells had an opposite effect

Because our previous study has found that ACTL6A can induce EMT in HCC, we further explored whether ACTL6A could induce EMT in colon cancer cells. First, we detected the expression of the classic EMT markers E-cadherin, vimentin and snail in ACTL6A-interfered SW480 and SW620 cells. Real-time PCR results showed that ACTL6A overexpression upregulated vimentin and snail mRNA and protein expression but downregulated E-cadherin expression in SW480 cells. In contrast, ACTL6A knockdown decreased vimentin and snail expression but increased E-cadherin expression in SW620 cells (Fig. 4a). Western-blot analysis showed similar results as the results of real-time PCR (Fig. 4b). We also observed the cell morphology using a phase contrast microscope, the images showed that SW620 cells with ACTL6A knockdown presented an oval or cobblestone epithelial morphology, while SW480 cells with ACTL6A overexpression presented an elongated cellular morphology and pseudopodia (Fig. 4c). The cell immunofluorescence for E-cadherin and Vimentin also indicated that when ACTL6A was knocked down, the SW620 cells showed stronger E-cadherin and weaker Vimentin fluorescence than did the control cells; while overexpression ACTL6A in SW480 cells showed the reverse results(Fig. 4d). Considering these results together, we suggest that ACTL6A might promote invasion and metastasis of colon cancer cells via inducing EMT.

## Discussion

Metastasis is the main cause of cancer-related deaths in colon cancer patients, thereby, it is crucial to illustrate the mechanism of metastasis. Existing studies and our previous study have indicated that ACTL6A participated in the metastasis of some cancer; however, the role of ACTL6A in colon cancer remains to be defined. In the present meaningful work, we found that ACTL6A expression was upregulated in colon cancer, its expression correlated with adverse clinicopathological features of colon cancer patients, and its overexpression promoted migration, invasion and EMT in colon cancer cells. These results provided the first description of the cancer-promoting and EMT activating role of ACTL6A in colon cancer.

EMT is a complex process that increases motility, invasiveness and degradation of the extracellular matrix (ECM) of cell; thus it broadly regulates the invasion and metastasis of cancer [13–15]. EMT has primitively been considered as the driving force of cancer cell dissemination, since then more studies have proven that EMT may participate in the whole process of invasion-metastasis cascade [13]. Solic and colleagues first found that colon cancer cells could undergo EMT when treated with EGF in 1995 [14]. Next, Brabletz and colleagues found that the EMT markers E-cadherin and β-catenin exhibited different expression patterns at the invasive front and central areas of the primary tumors in colorectal cancer in 2001, subsequently, accumulating evidences suggested that EMT occurred at the invasive front and produced single migratory cells to promote colon cancer metastasis, especially liver metastasis, and finally led to poor prognosis [16–18]. Thereby, more therapeutic strategies have aimed to target EMT in order to suppress the metastasis of colon cancer, such as gefitinib, withaferin A and Bortezomib [19].

In this study, we combined public databases and tissue samples from our center and found that ACTL6A expression levels in colon adenoma and cancer were both higher than that in normal colon, which first defined the expression pattern of ACTL6A in colon cancer. Next, analysis of the clinicopathological characteristics of colon cancer patients showed that ACTL6A expression positively correlated with advanced pT status, distant metastasis, poor differentiation and microvascular/perineural invasion. These data suggested that ACTL6Aexhibited a cancer-promoting role in colon cancer. Next, cytological experiments further confirmed that ACTL6A promoted colon cancer cell migration and invasion in vitro. These results were consistent with our previous study, which indicated that ACTL6A promoted HCC cells migration and invasion in vitro and in vivo [8]. Studies in SCC, glioma and rhabdomyosarcoma have also identified that ACTL6A promoted the cancer progression and metastasis, and related to poor prognosis [9–11]. According to these studies, we could strongly suggest that ACTL6A would play a pan-oncogenic role in cancer invasion and metastasis.

In this study we also found that ACTL6A promoted EMT in colon cancer cells. ACTL6A was formerly confirmed to participate in stemness maintenance and embryogenesis in stem/progenitor cells, in which EMT always plays a critical role [16, 20]. Additionally, studies have found that ACTL6A is important for proliferation and migration of stem/progenitor cells, which are also features of EMT [6, 16, 17]. Moreover, ACTL6A encodes an actin-related remodeling protein, and actin remodeling is essential for EMT-mediated cell-cell adhesion, invadopodia formation and cell morphological changes [18]. These evidences suggest that ACTL6A is a potential EMT-TF; additionally, our previous study first demonstrated that ACTL6A induced EMT in HCC [8]. Therefore, this study further verified the EMT activating role of ACTL6A, which expanded the understanding of the biological role of ACTL6A in cancer invasion and metastasis. However, the specific molecular mechanism of ACTL6A in inducing EMT is still obscure. Previous studies have indicated that ACTL6A was a cofactor of c-MYC and p63, which through activation of the Hippo-YAP pathway to promote proliferation and suppress differentiation of SCC and lead poor survival, both of c-MYC and p63 were also participants of EMT [9, 21]. The study in rhabdomyosarcoma demonstrated miR-206 was the upstream of ACTL6A, and miR-206 was identified to regulate EMT via PI3k/Akt/mTOR signaling, which was also the classical EMT related signaling [11, 22]. Our previous study in HCC found that ACTL6A activated Notch signaling, which was one of the most important signaling pathways in regulating EMT [8]. Taken all these researches, we could conclude that ACTL6A was closely connected with EMT in cancer. However, the exact regulatory mechanism of ACTL6A in promoting metastasis and EMT in colon cancer is still not fully studied; therefore, further studies are urgently needed in future. Nonetheless, our study indicated that ACTL6A was a new potential target for clinical therapeutic research in colon cancer.

## Conclusion

In summary, our findings indicated that ACTL6A exhibited pro-tumor function and acted as an EMT activator in colon cancer. ACTL6A may serve as a potential therapeutic target for colon cancer.

## Additional files

Additional file 1: The description of cell lines, MTT assay and plate colony formation assay. (DOCX 17 kb)

Additional file 2: **Figure S1.** (A) The ACTL6A interfered efficiency was assessed by real-time PCR, results showed that either overexpressed or knocked down plasmid had satisfied interfered efficiency and RNAi-1 had the strongest knockdown efficiency. (B) MTT assay showed that ACTL6A overexpression didn’t significant increased the proliferation rate of SW480 cells (*P* = 0.099), while ACTL6A knockdown slightly decreased the proliferation rate of SW620 cells (*P* < 0.05). (C) Plate colony formation assay showed that ACTL6A overexpression mildly increased the colony numbers of SW480 cells (*P* = 0.041), while ACTL6A knockdown had no significant effect in proliferation of SW620 cells (*P* = 0.074). (ZIP 256 kb)

## Abbreviations

ACTL6A: Actin-like 6A
cDNA: Complementary deoxyribonucleic acid
CSU: Central South University
DAB: Diaminobezidin 3, 3-diaminodiphenylamine
ECM: Extracellular matrix
EMT: Epithelial mesenchymal transition
EMT-TF: EMT-activating transcription factor
GEO: Gene Expression Omnibus
HCC: Hepatocellular carcinoma
HRP: Horseradish peroxidase
IHC: Immunohistochemistry
MMR: Mismatch repair
PBS: Phosphate-buffered saline
PVDF: Polyvinylidene fluoride
RNA: Ribonucleic acid
SCC: Squamous cell carcinoma
SWI/SNF: SWItch/Sucrose Non-Fermentable
