# Supplementary material for: ACTL6A expression promotes invasion, metastasis and epithelial mesenchymal transition of colon cancer
Source: BMC Cancer. 2018 Oct 22;18:1020. doi: 10.1186/s12885-018-4931-3 (PMC6198485; doi:10.1186/s12885-018-4931-3)
Supplement: Supplementary file 1 — The description of cell lines, MTT assay and plate colony formation assay. (DOCX 17 kb) [file 12885_2018_4931_MOESM1_ESM.docx]

**Supplementary Materials and Methods**

**Cell lines**

The human colon cancer cell lines SW480 and SW620 were kindly provided by Stem Cell Bank, Chinese Academy of Sciences (Shanghai, China) in July 2017. Next, the cell lines were authenticated by Short Tandem Repeat (STR) profiling following ISO 9001:2008 and ISO/IEC 17025:2005 quality standards by Genechem Co., Ltd (Shanghai, China) in October, 2017 (Supplementary File 1, 2).

**MTT assay**

The SW480 and SW620 cells were seeded into each well of 96-well plates at a density of 2 × 10^3^ cells/well respectively. Six wells of each group were detected every day. 100 μl fresh medium containing MTT (Sigma, St Louis, MO) with 0.5 mg/ml was put into each well and incubated at 37°C for 4 hours, then the medium was replaced by 100 μl of DMSO and shaken at room temperature for 10 min. The absorbance was measured and recorded at 490 nm by a spectrophotometer. All studies were conducted with 3 replicates.

**Plate colony formation assay**

For plate colony formation assay, cells were seeded into six-well culture dishes (Corning, NY) at a density of 5 × 10^2^ cells/dish and cultured for 2 weeks at 37°C and 5% humid CO2. The numbers of colonies per dish were counted under optical microscope after flushing with Phosphate buffer saline (PBS) followed by staining with 1% crystal violet. All studies were conducted with 3 replicates.
