# Supplementary material for: ACTL6A expression promotes invasion, metastasis and epithelial mesenchymal transition of colon cancer
Source: BMC Cancer. 2018 Oct 22;18:1020. doi: 10.1186/s12885-018-4931-3 (PMC6198485; doi:10.1186/s12885-018-4931-3)
Supplement: Supplementary file 2 — Figure S1. (A) The ACTL6A interfered efficiency was assessed by real-time PCR, results showed that either overexpressed or knocked down plasmid had satisfied interfered efficiency and RNAi-1 had the strongest knockdown efficiency. (B) MTT assay showed that ACTL6A overexpression didn’t significant increased the proliferation rate of SW480 cells (P = 0.099), while ACTL6A knockdown slightly decreased the proliferation rate of SW620 cells (P < 0.05). (C) Plate colony formation assay showed that ACTL6A overexpression mildly increased the colony numbers of SW480 cells (P = 0.041), while ACTL6A knockdown had no significant effect in proliferation of SW620 cells (P = 0.074). (ZIP 256 kb) [file 12885_2018_4931_MOESM2_ESM.zip › Supplementary Figure legends.docx]

**Supplementary Figure legends:**

**Figure S1:** (A) The ACTL6A interfered efficiency was assessed by real-time PCR, results showed that either overexpressed or knocked down plasmid had satisfied interfered efficiency and RNAi-1 had the strongest knockdown efficiency. (B) MTT assay showed that ACTL6A overexpression didn’t significant increased the proliferation rate of SW480 cells (*P* = 0.099), while ACTL6A knockdown slightly decreased the proliferation rate of SW620 cells (*P* < 0.05). (C) Plate colony formation assay showed that ACTL6A overexpression mildly increased the colony numbers of SW480 cells (*P* = 0.041), while ACTL6A knockdown had no significant effect in proliferation of SW620 cells (*P* = 0.074).
